# Clinical efficacy of tigecycline used as monotherapy or in combination regimens for complicated infections with documented involvement of multiresistant bacteria

**DOI:** 10.1007/s15010-014-0691-4

**Published:** 2014-11-04

**Authors:** W. R. Heizmann, P.-A. Löschmann, C. Eckmann, C. von Eiff, K.-F. Bodmann, C. Petrik

**Affiliations:** 1 Orgamed Laborsysteme GmbH, Maria-Schmid-Str. 14b, 94086 Bad Griesbach, Germany; 2 Pfizer Pharma GmbH, Linkstraße 10, 10785 Berlin, Germany; 3 Klinikum Peine, Virchowstraße 8 h, 31221 Peine, Germany; 4 Klinikum Barnim GmbH, Rudolf-Breitscheid-Straße 36, 16225 Eberswalde, Germany

**Keywords:** Tigecycline, Non-interventional study, Multiresistant pathogens, Methicillin-resistant *Staphylococcus aureus*, Extended-spectrum beta-lactamase, Vancomycin-resistant enterococci

## Abstract

**Introduction:**

Tigecycline is an established treatment option for infections with multiresistant bacteria (MRB). It retains activity against many strains with limited susceptibility to other antibiotics. Efficacy and safety of tigecycline as monotherapy or in combination regimens were investigated in a prospective noninterventional study involving 1,025 severely ill patients in clinical routine at 137 German hospitals.

**Materials and methods:**

Data on the full population have been published; our present analysis focuses on infections caused by MRB. The study population included patients with complicated infections, high disease severity (APACHE II > 15: 65 %) and high MRB prevalence. Most patients had comorbidities, including cardiovascular disease, renal insufficiency, and/or diabetes mellitus. Treatment success was defined as cure/improvement without requirement of further antibiotic therapy.

**Results:**

Pathogens isolated from 215 evaluable patients with documented MRB infections included 132 methicillin-resistant *Staphylococcus aureus* (MRSA), 42 vancomycin-resistant *Enterococci* (VRE) and 67 Gram-negative extended beta-lactamase (ESBL) producers. Of the MRB subpopulation, 140 patients received tigecycline monotherapy, 75 were treated with combination regimens. High overall clinical success rates were recorded for MRB infections treated with tigecycline alone (94 %) or in combinations (88 %); in detail intraabdominal infections (monotherapy: 90 %; combinations: 93 %), skin/soft tissue infections (93; 100 %), community-acquired pneumonia (100; 100 %), hospital-acquired pneumonia (94,7; 72,7 %), diabetic foot infections (89; 33 %), blood stream infections (100; 100 %) and multiple-site infections (92; 71 %).

**Conclusions:**

Tigecycline achieved high clinical success rates in patients with documented infections involving MRB strains despite high disease severity. These results add to the evidence indicating that tigecycline is a valuable therapeutic option for complicated infections in severely ill patients with a high likelihood of multidrug-resistant pathogen involvement.

## Purpose

Tigecycline is a glycylcycline antibiotic with a broad spectrum of antimicrobial activity covering bacteria with resistance against multiple antibiotics (MRB) such as vancomycin-resistant enterococci (VRE), methicillin-resistant *Staphylococcus aureus* (MRSA), extended-spectrum beta-lactamase producing Enterobacteriaceae (ESBL) and strains of the *Acinetobacter baumannii* group [1–4].

In the US [5] and Europe [6], tigecycline is approved for the treatment of complicated intraabdominal infections (cIAI) and complicated skin and skin tissue infections (cSSTI). In the US, tigecycline is also indicated for community-acquired bacterial pneumonia.

The patient population in the two pivotal phase III studies on tigecycline in cIAI had a relatively low mean initial APACHE II score of 6.3, as patients with APACHE II scores >30 were excluded [7]. The number of severely ill patients was limited in both phase III cSSTI trials as well [8]. Thus, published data from prospective (comparative) trials on tigecycline used in higher risk patients with complicated, pre-treated infections and high risk of drug-resistant pathogens are limited.

Most data on tigecycline in severely ill patients are derived from retrospective analyses or studies focused on identified pathogens rather than clinical syndromes. Bassetti et al. [9] reported on a single-center prospective observational study of tigecycline in severely ill patients with various complicated infections. The authors found high response rates for peritonitis, cSSTI and blood stream infections despite unfavorable patient risk profiles (mean APACHE II score 21; high prevalence of severe comorbidities).

In recent years, Enterobacteriaceae developed a range of antimicrobial resistances that reduce therapeutic choices to a very limited set of active antibiotics. The spread of ESBL-producers and bacterial strains expressing carbapenemases is causing much concern on the future options of effective antibacterial therapy in hospitals [10]. In addition, in the Gram-positive spectrum of pathogens, MRSA remains a threat in cSSTI [11], DFI [12], and hospital-acquired pneumonia (HAP) [13], while VRE are commonly implicated in severe cIAI and blood stream infections (BSI) [14]. Because these pathogens are commonly found in infections taking a severe course, tigecycline becomes an increasingly important treatment option for a broad range of severe infections, particularly as empirical therapy in patients at risk for MRB.

Consequently, there is a need for additional clinical data to evaluate the usefulness of tigecycline, thereby providing additional evidence for the rational and safe use of this antibiotic.

This sub-analysis of a prospective, non-interventional study investigated the efficacy and safety of tigecycline used alone or in combination in the real-life hospital setting in Germany. Results obtained in the total patient population have been published before [15]. Here, we present data on patients suffering from infections with documented involvement of bacteria exhibiting multidrug-resistant phenotypes. We characterized the subpopulation treated with tigecycline for these infections in various indications and determined treatment outcomes associated with tigecycline used alone or in combination with other antimicrobials.

## Methods

Details on the methodology of the non-interventional parent study have been published before [15]. Briefly, hospital-based physicians prospectively documented data on patients treated with tigecycline for cIAI, cSSTI or other severe infections according to local routine practice. The population observed in this study included severely ill patients with previous/failed antimicrobial treatment and/or involvement of drug-resistant pathogens. Infections were classified as hospital vs. community acquired depending on the first manifestation of the infection after or before 48 h of hospitalization. The study protocol involved an initial intravenous dose of 100 mg of tigecycline (Tygacil^®^; Pfizer Pharma GmbH, Berlin, Germany), followed by 50 mg tigecycline every 12 h, as recommended in the product label.

The present analyses include only those patients who had infections with documented involvement of multiresistant bacteria (VRE, MRSA, ESBL-producers) and evaluable treatment outcomes. ESBL-production was detected by combination disk testing with clavulanic acid or VITEK II, methicillin resistance by testing cefoxitin and VRE by E-test, breakpoint agars, or VITEK II according to the protocol of the local microbiology laboratory.

The investigators rated the therapeutic outcome as cure, improvement with no further need for antibiotic treatment, failure to respond or not evaluable. Rating the outcome as cure required full resolution of symptoms of infection, whereas improvement was defined as significant improvement of symptoms but without complete resolution of infection. Outcome was rated 1–3 days after the end of tigecycline therapy or at hospital discharge. Treatment success was defined as cure or improvement with no further need for antibiotic treatment.

## Results

### Patient demographic and clinical characteristics

1025 patients were treated with tigecycline in 137 German hospitals. Of these patients, 256 had infections due to multiresistant bacteria. Demographic and clinical characteristics of evaluable patients are presented in Table 1.

**Table 1:** Patient demographics, comorbidities and severity scores at baseline

Patient demographics	Total population	Patients with MRB
Number of patients, *n*	1,025	256
Demographic characteristics		
Male, % (*n*)	62.8 % (642)	64.8 % (166)
Age, mean years ± SD (range)	64.4 ± 13.7 (18–94)	66.5 ± 12.0 (19–88)
Clinical characteristics		
BMI, mean, kg/m^2^ ± SD (range)	27.7 ± 6.5 (14–90)	28.4 ± 7.0 (14.6–58.8)
Treatment on ICU, % (*n*)	53.2 % (545)	41.8 % (107)
History of prior antibiotics, % (*n*)	84.5 % (864)	83.2 % (213)
Comorbidity, % (*n*)	96.5 % (989)	96.1 % (246)
APACHE II score >15, % (*n*)	64.9 % (607)	68.4 % (162)
APACHE II score, mean (median)	18.8 (18.0)	19.4 (19.0)
Patients with treatment on ICU	20.0 (20.0)	21.4 (21.0)
Patients with treatment outside ICU	17.3 (17.0)	17.9 (18.0)

A large proportion of this predominantly elderly MRB subpopulation (mean age: 66.5 years) was treated on intensive care units (41.8 %). The median APACHE II score was 21.5. Virtually all patients had at least one comorbidity. Most patients (83.2 %) had received prior therapy with other antibiotics.

### Pathogens and sites of infection

MRSA was the most commonly isolated multiresistant pathogen (61.4 % of the patients), followed by ESBL-producers (31.2 %) and VRE (19.5 %) (Table 2). Most patients with MRB had cIAI (32.6 %) or cSSTI (25.6 %), followed by other severe infections, such as hospital- or community-acquired pneumonia (20.0 %), diabetic foot infections (DFI; 14.0 %), blood stream infections BSI (10.2 %) or multiple-site infections (MSI; 12.6 %). MRSA was the predominant pathogen in patients with cSSTI (90.9 %), CAP (84.6 %), HAP (70.0 %), DFI (100 %) and BSI (68.2 %). ESBL-producers were the most common MRB in patients with cIAI (50.0 %) and VRE were the second most pathogens isolated in patients with cIAI (38.6 %).

**Table 2:** Distribution of multiresistant bacteria by site of infection (patients with evaluable treatment outcome; *n* = 215)

Drug-resistance phenotype, % (*n*)	Patients with documented MRB infection			
	Any MRB	VRE	MRSA	ESBL
Total MRB population	100 % (215)	19.5 % (42)	61.4 % (132)	31.2 % (67)
Intraabdominal infection (cIAI)	32.6 % (70)	38.6 % (27)	27.1 % (19)	50.0 % (35)
Skin and soft tissue infection (cSSTI)	25.6 % (55)	5.5 % (3)	90.9 % (50)	7.3 % (4)
Diabetic foot infection (DFI)	14.0 % (30)	−(0)	100.0 % (30)	10.0 % (3)
Community-acquired pneumonia (CAP)	6.0 % (13)	7.7 % (1)	84.6 % (11)	38.5 % (5)
Hospital-acquired pneumonia (HAP)	14.0 % (30)	−(0)	70.0 % (21)	30.0 % (9)
Blood stream infection (BSI)	10.2 % (22)	18.2 % (4)	68.2 % (15)	36.4 % (8)
Multiple-site infection (MSI)	12.6 % (27)	14.8 % (4)	63.0 % (17)	44.4 % (12)

Patients could have more than one MRB

### Mode and duration of therapy

The great majority of patients (initial dose ≥95.7 %; maintenance doses ≥91.9 %) received tigecycline at the recommended dosage^1^ as monotherapy (65.1 %) or in combination regimens (34.9 %) (Table 3). Combination therapy was most common in cIAI (40.0 %). Most patients treated with combination regimens received ceftazidime, a carbapenem, a fluoroquinolone or metronidazole in addition to tigecycline (Table 4).

**Table 3:** Mode of therapy in patients with multiresistant pathogens by site of infection (patients with evaluable treatment outcome; *n* = 215)

Proportion of patients, % (*n*)	Monotherapy	Combination therapy
Total MRB population	65.1 % (140)	34.9 % (75)
Intraabdominal infection (cIAI)	28.6 % (40)	40.0 % (30)
Skin and soft tissue infection (cSSTI)	31.4 % (44)	14.7 % (11)
Diabetic foot infection (DFI)	19.3 % (27)	4.0 % (3)
Community-acquired pneumonia (CAP)	8.6 % (12)	1.3 % (1)
Hospital-acquired pneumonia (HAP)	13.6 % (19)	14.7 % (11)
Blood stream infection (BSI)	12.9 % (18)	5.3 % (4)
Multiple-site infection (MSI)	9.3 % (13)	18.7 % (14)

Patients could have more than one MRB; 2 patients with HAP were diagnosed with BSI as well, these were not categorized as MSI because the lung infection was regarded as the focus of BSI

**Table 4:** Antibiotics most commonly administered in combination with tigecycline (patients with evaluable treatment outcome; *n* = 215)

Antibiotic agent	Patients, % (*n*)
All agents	34.9 %(75)
Ceftazidime	11.2 %(24)
Carbapenem (meropenem, imipenem)	5.6 %(12)
Fluoroquinolone (ciprofloxacin, levofloxacin)	4.7 %(10)
Metronidazole	3.7 % (8)
Piperacillin (±tazobactam)	1.9 % (4)
Vancomycin	1.4 % (3)
Cefepime	1.4 % (3)
Sulbactam	0.9 % (2)
Clindamycin	0.9 % (2)
Gentamicin	0.9 % (2)
Others	2.3 % (5)

Median treatment duration was 8 days for BSI (range 4–17), 9 days for HAP (5–17), 10 days for IAI (2–40) and CAP (7–15), 11 days for SSTI (4–33) and DFI (4–42), and 12 days for MSI (5–42).

### Clinical outcome

The clinical outcome of tigecycline treatment per patient subgroup is shown in Tables 5 and 6. Treatment success rates were generally in the range of 80–100 % regardless of the type of involved MRB.

**Table 5:** Treatment success rates (cure + improvement) in patients with multiresistant pathogens by drug-resistance phenotype (patients with evaluable treatment outcome; *n* = 215)

Treatment success % (*n*/*N*)	Patients with documented MRB infection			
	Any MRB	VRE	MRSA	ESBL
Total MRB population	91.6 % (197/215)	97.6 % (41/42)	90.2 % (120/132)	91.0 % (61/67)
Intraabdominal infection (cIAI)	91.4 % (64/70)	96.3 % (26/27)	89.5 % (17/19)	91.4 % (32/35)
Skin and soft tissue infection (cSSTI)	94.5 % (52/55)	100 % (3/3)	94.0 % (47/50)	100 % (4/4)
Diabetic foot infection (DFI)	83.3 % (25/30)	−(0/0)	83.3 % (25/30)	66.6 % (2/3)
Community-acquired pneumonia (CAP)	100 % (13/13)	100 % (1/1)	100 % (11/11)	100 % (5/5)
Hospital-acquired pneumonia (HAP)	86.7 % (26/30)	−(0/0)	85.7 % (18/21)	88.8 % (8/9)
Blood stream infection (BSI)	100 % (22/22)	100 % (4/4)	100 % (15/15)	100 % (8/8)
Multiple-site infection (MSI)	81.5 % (22/27)	100 % (4/4)	76.5 % (13/17)	83.3 % (10/12)

Patients could have more than one MRB

**Table 6:** Treatment success rates (cure + improvement) in patients with multiresistant pathogens by mode of therapy (patients with evaluable outcome; *n* = 215)

Treatment success % (*n*/*N*)	Monotherapy	Combination therapy
Total MRB population	93.6 % (131/140)	88.0 % (66/75)
Intraabdominal infection (sIAI)	90.0 % (36/40)	93.3 % (28/30)
Skin and soft tissue infection (cSSTI)	93.2 % (41/44)	100 % (11/11)
Diabetic foot infection (DFI)	88.9 % (24/27)	33.3 % (1/3)
Community-acquired pneumonia (CAP)	100 % (12/12)	100 % (1/1)
Hospital-acquired pneumonia (HAP)	94.7 % (18/19)	72.7 % (8/11)
Blood stream infection (BSI)	100 % (18/18)	100 % (4/4)
Multiple-site infection (MSI)	92.3 % (12/13)	71.4 % (10/14)

Complicated IAI were successfully treated in 91.4–96.3 %. HAP success rates were somewhat higher with MRSA (94.7 %) than with ESBL-producers (88.8 %), but the patient number was low in the latter subgroup. Tigecycline was effective in most DFI (83.3 %) which were almost exclusively caused by MRSA. In cSSTI, again dominated by MRSA, an overall success rate of 94.5 % was observed. The treatment success rate was 100 % for BSI and CAP in all MRB subgroups. Patients with multiple-site MRB infections had a success rate of 81.5 %.

**Fig. 1:**
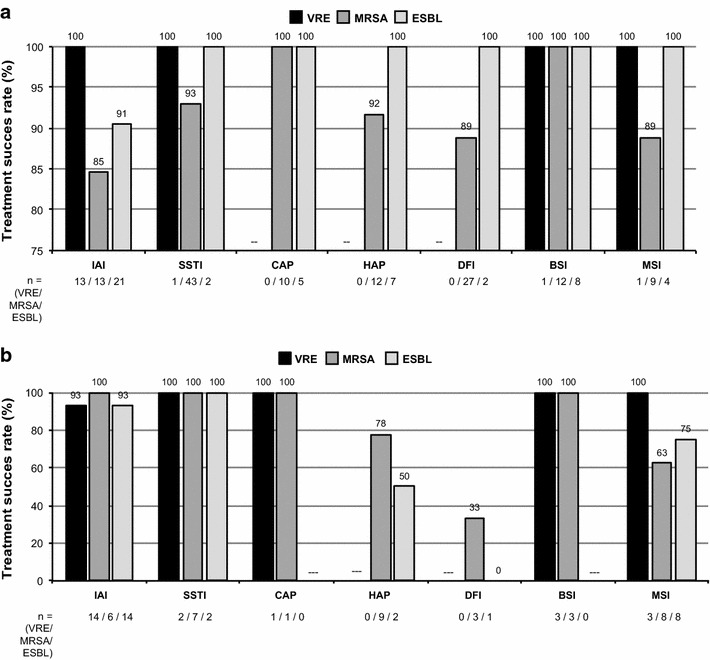
Treatment success rates (cure + improvement) in patients with multiresistant pathogens **a** Monotherapy, **b** combination therapy. *IAI* intraabdominal infection, *BSI* blood stream infection, *CAP* community-acquired pneumonia, *DFI* diabetic foot infection, *HAP* hospital-acquired pneumonia, *MSI* multiple-site infection, *SSTI* skin and soft tissue infection

Regarding treatment modality, the success rate was 93.6 % for monotherapy, with rates ranging from 89.9 to 100 % for the different types of infection and drug-resistant bacteria (Fig. 1a; Table 6).

Using combination therapies, 88.0 % of the patients were successfully treated, with rates ranging from 71.4 % to 100 % for all types of infections. The rate was lower in DFI (33.3 %), albeit based on only 3 patients in this subgroup (Fig. 1b; Table 6).

## Discussion

The patients analyzed in this subpopulation with MRB infections were treated with tigecycline in routine settings in German hospitals. They suffered from complicated IAI, SSTI, and/or other severe infections involving multiresistant bacteria.

Rates of clinical cure or improvement were high in this subpopulation. A total of 91.6 % of patients with MRB infections were successfully treated, 93.6 % with monotherapy and 88.0 % with tigecycline combinations.

Success rates for monotherapy were consistently higher than 90 % for all multiresistant pathogens and higher than 80 % for all disease types; rates were particularly high in BSI (100 %), CAP (100 %) and cSSTI (95 %).

The success rates tended to be somewhat lower in combination therapies (88.0 vs. 93.6 % with monotherapy), mostly due to the response rates in patients with MSI (71.4 %), or HAP (72.3 %). This divergence may have been caused (1) by a higher likelihood of combination therapies being used in patients with higher disease severity, (2) the choice of the combination drug, (3) random effects due to small patient numbers, and (4) the likelihood of higher morbidity in patients with infection at multiple sites of infection.

Conversely, the treatment success rate of MRB nosocomial pneumonia was 94.7 % in patients receiving tigecycline monotherapy at standard dosage. This is a reassuringly high rate in the light of data obtained in the phase III HAP study of tigecycline versus imipenem that failed to confirm the non-inferiority of tigecycline in the clinically evaluable patient subset. A subsequent phase II study with tigecycline used at higher dosages indicated increased efficacy with a clinical cure rate of 85 % [16]. There are several, at least, theoretical reasons why non-bactericidal antimicrobial agents such as tigecycline are effective in severe infections [17].

The limitations of this study include its non-controlled observational design that may be associated with several biases and uncertainties, and the lack of rigorous criteria of diagnosis and assessment of response. Despite these shortcomings, this analysis of a sizeable sample of patients with severe MRB infections provides evidence of the usefulness of tigecycline in this diverse and difficult-to-treat population.

Non-interventional studies provide insights into the real-life utility of antibiotics beyond the preselected cohorts treated in randomized trials. Despite that patients infected with multiresistant bacteria are not excluded from pivotal trials, they usually do not represent a large proportion of the whole patient population. Observational studies are particularly useful for the evaluation of substances that are used in indications and situations outside the scope covered by pivotal trials, e.g., in patients with high-risk profiles, multiple comorbidities, highly resistant pathogens, extensively pre-treated infections [18].

## Conclusions

Our subpopulation analysis of the prospective tigecycline non-interventional study conducted in routine settings confirmed the efficacy of tigecycline in the treatment of severely ill patients with complicated, mostly pre-treated infections involving multidrug-resistant pathogens. Tigecycline was administered at the recommended dose with few exceptions.

Tigecycline used alone or in combination was highly effective against infections caused by multidrug-resistant Gram-positive and Gram-negative pathogens in patients even with high disease severity.

These results add to the accumulating evidence indicating that tigecycline is a valuable therapeutic option for complicated infections in severely ill patients at high risk of the involvement of multidrug-resistant pathogens.

## Footnotes

1 Initial dose was not reported in 1 patient; maintenance dose was not reported in 10 patients. The following divergent dosage regimens were reported: initial dose: 50 mg (7 patients), 70 mg (1), 2 x 100 mg (1), not specified (1); maintenance doses: 25 mg twice daily (1); 25 mg twice weekly (1); 50 mg once daily (5); 100 mg once daily (1) 100 mg twice daily (1), 150 mg twice daily (1); not specified (1).
